# Developing a gender-based approach to chronic conditions and women’s health: a qualitative investigation of community-dwelling women and service provider perspectives

**DOI:** 10.1186/s12905-015-0264-4

**Published:** 2015-11-21

**Authors:** Michelle DiGiacomo, Anna Green, Emma Rodrigues, Kathryn Mulligan, Patricia M. Davidson

**Affiliations:** Faculty of Health, Centre for Cardiovascular and Chronic Care, Broadway, University of Technology Sydney, Sydney, NSW 2007 Australia; Illawarra Shoalhaven Local Health District, Women’s Health Service, Warrawong, 2502 NSW Australia; School of Nursing, Johns Hopkins University, Baltimore, MD 21205 USA

**Keywords:** Gendered approach, Chronic disease, Women’s health

## Abstract

**Background:**

Chronic conditions contribute to over 70 % of Australia’s total disease burden, and this is set to increase to 80 % by 2020. Women’s greater longevity means that they are more likely than men to live with disability and have unique health concerns related to their gender based roles in society. Cultural and social issues can impact on women's health and are important to consider in health services planning and research. In this study, we aimed to identify barriers and facilitators to providing a gender-based approach to chronic conditions and women's health in an eastern metropolitan region of Australia.

**Methods:**

Focus groups were used to engage both community-dwelling women who had chronic conditions and relevant professional stakeholders in the target area. Recorded proceedings underwent thematic analysis.

**Results:**

Five focus groups were conducted with professional stakeholders and women community members in February and March 2014. Resultant themes included: women’s disempowerment through interactions with health systems; social and economic constraints and caregiving roles act to exclude women from participating in self-care and society; and empowerment can be achieved through integrated models of care that facilitate voice and enable communication and engagement.

**Conclusions:**

This study underscores the importance of including perspectives of sex and gender in health care services planning. Tailoring services to socio-demographic and cultural groups is critical in promoting access to health care services. Unique epidemiological trends, particularly the ageing of women and new migrant groups, require particular attention.

## Background

Chronic conditions contribute to over 70 % of Australia’s total disease burden, and this is set to increase to 80 % by 2020. As women live longer than men, they often do so with greater disability [1, 2]. Women are also more likely to have unique health concerns and issues related to their gender roles in society. Cultural and social issues also impact on women's health and are important to consider in health services planning and research. In women from socio-economically deprived groups and in culturally and linguistically diverse (CALD) groups, needs are likely to be greater and unique to social, economic, and cultural circumstances. As women assume a greater amount of informal caregiving, this can also alter how they cope and adjust to living with a chronic illness [3].

Traditionally, health systems have been built around responding to acute needs and episodic health problems rather than chronic conditions. The current and projected incidence of chronic disease means that there is an urgent need to change how health services are delivered. As the majority of this care is provided in the community, it is important to consider the gender-based implications of such an approach. To date, there has been a limited focus on the needs of women and how social determinants and socio-cultural factors impact on their wellbeing. This has meant that women with such conditions have not received the level of support and services needed to ensure the best possible outcomes [4].

For the purposes of this study, we refer to women’s health issues as “*social conditions, illnesses, and disorders unique to, more prevalent among, or more serious in women or for which there are different risk factors, interventions, or strategies for women than for men*.” [5] Gender is a dynamic and socially defined construct comprising role enactment, values, and beliefs of both men and women. Gender-based roles, enactment and health outcomes are shaped by social, economic, political and cultural factors, rather than by biology alone. Social determinants of health are the economic and social conditions under which people live that determine health and well-being. These determinants, such as low levels of education and poverty, can set a course for a lifetime of hardship and disability. These factors have a significant impact on how people view the world and how they enact health-seeking behaviors. In addition to social and economic roles, the capacity of women to actively engage in decision-making and access to resources can impact on their health and well-being [6, 7].

Women continue to experience inferior health outcomes across a number of conditions, despite human rights advances and average longevity in many developed countries as surpassing men by several years. The launch of UN Women, the United Nations entity for gender equality and the empowerment of women, on February 24, 2011, was an important signal of the need to focus on the unique needs of women and the influence this has on societal outcomes [7].

Peak health bodies have called for gender-based research to inform health service planning and delivery due to the gap in understanding of how the experience of chronic conditions differs between men and women [8]. From the available research that focuses on women’s chronic care experiences within health care systems, we have learned that barriers to women obtaining recommended care include having a chronic disease [9], experiencing psychological distress [10], or having negative experiences in interactions with health services [11, 12], stigmatisation [13], or having family roles that compete for time and resources [14, 15]. Other literature states that health care systems can be disabling for women by positioning them as the ill ‘other’ [16], compelling presentation as a ‘credible patient’ [17], and imposing cultural notions of the female body from positions of power [13].

This paper reports on a qualitative study that was situated within a larger health services project set in an eastern metropolitan region of Australia. The health service set out to develop policy, practice, and research recommendations to better address the needs of women with chronic conditions. To do so, it was necessary to ascertain experiences and perspectives of both the women in that community and health service personnel. In this qualitative study, we aimed to identify barriers and facilitators to providing a gender-based approach to chronic conditions and women's health.

## Methods

This project was set in the Illawarra region south of Sydney in New South Wales, Australia. This region has a population approaching half a million people of whom approximately 25 % were born overseas, 14.4 % speak a language other than English at home, 13 % are informal caregivers, and 28 % are aged 55 and over, of whom approximately half are women [18].

We sought to ascertain women's experiences with the health care system, particularly in relation to having a chronic condition, as these voices have thus far been under-represented in the literature. Mead [19] has asserted that only by involving women in the planning for services will you gain their engagement. By including women’s voices in this study, they were able to contribute in a significant and meaningful way to health service planning [20]. In addition, we sought stakeholder perspectives of these issues as they would have witnessed or been a part of women’s journeys throughout the health system. Participation in the study involved separate discussions with groups of women in the community and professional stakeholders about key issues in women’s health relevant to chronic illness, barriers and facilitators in managing conditions, and experiences of interfacing with the health system. Participants were asked to draw on their professional or personal experience to provide a description of women’s journeys through the health care system, ways in which being female may influence living with chronic conditions, and what resources might assist fostering a gendered approach to health care. Focus groups were chosen to elicit perspectives both from individual participants and their responses to others’ perspectives in the group. For example, participants’ stories prompted discussion within the groups of similar or different examples. Each focus group lasted approximately one hour. Two experienced university researchers with social science backgrounds facilitated the focus groups. The researchers had no relationship with any of the participants. With permission of participants, the group discussions were recorded and later transcribed for analysis. Pseudonyms replaced participants names prior to analysis and other identifying features were removed following analysis. Focus groups were held in private conference rooms at a local hospital (professional groups), the town library, and a local women’s health centre (community groups) to maximise access for women wishing to participate.

We used purposive sampling methods to include diverse, informed opinions in the focus groups. Participants attending local community health centres and other relevant health facilities across the health service catchment area were informed of community consultations taking place through a promotional poster advertising the dates and locations of each session. The poster invited participation from women who had experienced a chronic illness. Key stakeholders working in chronic and complex care in both government and non-government organisations in the target area were identified by a research team member who was employed in a women’s health position. The researcher distributed study advertisements via email to all identified personnel. Potential participants contacted the research team to express their interest and were given an information sheet and consent form. Prior to beginning data collection, participants were given the opportunity to ask any questions about the study and requirements of their involvement. Following any clarification, participants signed consent forms. Approval to conduct this project was granted by the University of Technology Sydney (EC00146), the University of Wollongong (HE13/375), and the lllawarra Shoalhaven Local Health District (ISLHD) Human Research Ethics Committees.

Data analysis followed the iterative mode of allowing ongoing preliminary analysis in writing up observations of focus group sessions. Upon transcription of audio recordings of proceedings, a general inductive analysis [21] began with close reading of the raw data and note-taking to facilitate immersion. Notes were made on a line-by-line basis throughout each transcript as described by Minichiello [22]. Data were coded and clustered into related categories and compared within and across groups. Themes were inductively derived from the data. Steps taken to maximise reflexivity and analytical rigour included using multiple independent coders and discussions within the research team to reach agreement. Although data were collected and analysed according to participant classification as either professional stakeholder (PS) or community member (CM), similarities across emergent themes indicated appropriateness of integration.

## Results

Two focus groups were conducted with professional stakeholders in February 2014, comprised of 6 female participants who were currently employed in chronic care, multicultural health, and drug and alcohol areas within the region. In addition, three focus groups were held in March 2014 with 14 female community members who reflected on their or others’ experiences of the health care system in relation to chronic conditions.

A prominent theme centered on women’s disempowerment and loss of voice through communication and interactions within the health system. Sub-themes included perceptions of being dismissed and avoided by health professionals, disempowerment around communicating with health professionals, and misperceptions around understanding and language proficiency in chronic conditions, which had particular implications for older CALD women. A second theme involved the impact of social and economic constraints and caregiving roles in excluding women from participating in self-care and society, more broadly. A third theme highlighted services and providers that facilitate voice and enable communication and engagement as empowering women with chronic conditions.

### Loss of voice and disempowerment in communication and interactions with health professionals

Participants shared several examples of women’s interface with health professionals that were ascribed as influencing women’s use of and interactions within the health system. Women’s interactions with health professionals were described as being affected by a range of individual, provider, socioeconomic, and cultural factors. These included: doctors attributing presentations to mental health issues, concerns over labeling and stigma, generational and cultural communication and socialization norms, Anglo-dominated world views and language, education level, time restrictions and comorbidities, and misconceptions of English language mastery.

#### Women felt dismissed and avoided by health professionals

One participant recounted the complexities and implications associated with having multiple chronic conditions, which was described as ‘the norm’. The following excerpt illustrates her perspective of how help-seeking was mismanaged by a provider:

*PS: “Multiple chronic illnesses means they’ve got to visit multiple services. Multiple services means multiple bus trips, taxi trips…Um, changing their lifestyle…recently a lady who is a long-term diabetic saw her GP and needs a podiatrist. Well, they don’t have a podiatrist there anymore, and that was the end of it. There was no, ‘But we’ll you refer you to - we’ll look into’- anything. ‘We don’t have them anymore. So tough luck.’ Yeah, she’s on the chronic disease plan, but they don’t have a podiatrist. And so, rather than refer her, it was just the end of the story.*

*Moderator: Why do you think that happened?*

*PS: Um, I think it seemed - from talking to her, I think she’s a ‘frequent flyer’, and I think he’s probably fed up. So, they start to get that attitude, which is extremely difficult not to get…but still she needs a podiatrist. They do become ‘frequent flyers’ and the doctors have got 15 minutes.*

*Moderator: Are women viewed more negatively if they’re a ‘frequent flyer’?*

*PS: …I’m just thinking if that was a man that had been asking for a podiatrist? Yeah. I do think they - they put women into the ‘nut’ category, yeah. She was told she was a hypochondriac and I don’t think she is. I think she’s just concerned with her health. She’s a very smart lady. I just think she challenges this particular GP, and he doesn’t like it. So, I think that’s what’s going on there. So we’re trying to find her another GP who doesn’t mind being challenged. But, this is a lady who will stand up for herself. She’s won, but it’s taken her months.”(PS)*

Illustrated in this excerpt is the complexity associated with the management of multiple chronic conditions and how it is potentially influenced by beliefs and attitudes of health professionals. Because of this woman’s tenacity and assertiveness, she eventually found support to get the care she needed to manage her conditions. Dismissal and attribution of concerns to mental health problems was highlighted also in a discussion about some cultural groups’ health beliefs and the normalizing of chronic conditions.

Another professional stakeholder participant discussed that the women she works with may avoid seeking help or may lie to minimize financial shortfalls when unable to refill prescriptions for fear of being labeled and stigmatized. Community participants described their perception that doctors are disbelieving or dismissive of their health complaints, particularly in the case of chronic conditions. In one community member focus group, women discussed the feeling of being dismissed by health professionals and not having concerns addressed:

*“I think the biggest problem that I have come across with severe pain is the actual patient being believed…My mother-in-law suffered for 40 years and she’s still not being believed…I think, to be believed is one of the biggest problems…Not only do they still not believe her, but they try and give her something so that she will be asleep today instead of her being awake.” (CM)*

*“well last time, say for instance, you gave me Panadol and I come again to you and this time I get Panadol but in a different package. In a way of saying, well take this, go home and…don’t stay here. Don’t bother me. Go home [laughter].” (CM)*

These excerpts depict the women’s perception of being dismissed by doctors who perhaps do not appraise their complaints as requiring investigation. They perceived the medications offered as attempts to appease and silence. The following women discussed a shared experience of feeling ashamed for having sought medical advice:

*CM1: “I have been going for same thing for 15 years. I have a sore left leg; it’s constant. If it’s not sore, it’s cold. But it’s like, I’m going there and I’m whinging. And I actually feel sometimes that I am whinging, so I don’t go again.” (CM)*

*CM2: “Yeah, but when you go and see a professional person, and you come out of it feeling like you shouldn’t have gone, something’s wrong.” (CM)*

*CM1: “I feel, like, when I go to the specialist or when I go to have an X-ray they must ring up and say, she’s coming [laughs]. Probably don’t do that, but that’s how I sometimes feel.” (CM)*

#### Women’s disempowerment around communicating with health professionals

Participants perceived that a source of women’s disempowerment was the dominance of men in health professional positions, particularly in specialty fields, but also working in health facilities, in general. When asked to elaborate on what was meant by disempowerment, one participant related it to not seeking second opinions. Reasons for this were multifaceted and may reflect cultural, geographic, logistic, financial, transport, and caregiver role barriers. Disempowerment was also discussed in relation to inhibited dialogue with health professionals. For example, although women may be perceived as more proactive, older generations may feel that the ‘doctor knows best’ and accept his or her word without question. Depending on the doctor-patient rapport and individual characteristics and skills, women may not feel confident to ask questions during consultations. Having an advocate who can interpret what is being said was described as useful in taking in so much new information. The excerpt below provides an example of women’s backgrounds and experiences as influencing their interactions with medical professionals:

*“…older women treat doctors like little gods up to a point. Some don’t; it depends where they come from and what their situation is, I guess. You know, some of those women are very strong, but, you know, when they go to a doctor, it’s ‘yes doctor,’ I think they’re used to kowtowing to people in authority, generally” (CM)*

*“they don’t think they’re worthy of, you know, much consideration either. Like, with someone like me, who’s got the background that I’ve got, I’m used to trying to get what I want out of the medical system. And I will pursue my goals and get them to fit in with me and do what I want and give me a referral to someone if I want it.” (CM)*

As noted in this excerpt, interactions depend heavily on the characteristics and skills of people involved. Participants agreed that having frank discussions with medical teams is ‘healthy’, yet some believed women who are less assertive may have more difficulty obtaining information. Older female patients did not always feel comfortable broaching topics such as incontinence and menopause. The following excerpt depicts one former health worker’s experiences working in a women’s health clinic:

*“Women used to come in and tell us things, a lot of things, you know, about their local doctors and how they didn’t listen to them, that’s a major one. And how they just pushed them off as long as they got them out of the place in five minutes…they would write a script, especially with women with menopause, they were the main complainants…male doctors didn’t want to know about it, basically…even said things like, ‘I can’t help you with that’, if women were suffering prolapses or anything like that.” (CM)*

*“Let’s face it, if you've got a female GP, you’re lucky really…I find women are better at listening than men” (CM)*

Avoidance of personal health topics meant that some women often went several years without needed pain-relieving treatment. Lack of understanding and not seeking clarification from doctors was discussed in regards to some CALD women feeling intimidated in consultations with health professionals. They may convey understanding, but this was explained as an avoidance strategy:

*“[Women] will sit there and just go, ‘yes’, and not take the tablets, because they don’t know what it’s about. But they tell the doctor they do, because it’s easier…and because it’s intimidating.” (PS)*

#### Misperceptions around understanding and language proficiency in chronic conditions

Health professionals may erroneously assume chronic disease sufferers or their caregivers, including those who may be health professionals, have acquired expertise and capacity for self-management throughout the duration of illnesses.

*“The more chronic the disease, the less…interaction they get. So the less time people are wanting to spend to try to explain things, especially now, because you've had it for 10 years… So, ‘I don’t have to explain it, because you’ve had it for 10 years’…Because it’s a function of self-management isn’t it? And it happens whether you speak English or not.” (PS)*

Also noted were misconceptions around English language proficiency. Although some people from non-English speaking backgrounds appear to understand and communicate in English reasonably well, this was not always an accurate representation:

*“One of the barriers is around identifying what communication issues are. And that’s variable. So staff will think they can talk well with this person, because it’s broken English.” (PS)*

*So that’s a big issue for the women in our community, because when they get older, not only do they suffer the pain, but they also forget the English…it is difficult for her to communicate now because she’s forgotten the English.” (CM)*

In light of this finding, health professionals may need to take steps to ensure understanding, such as involving an interpreter, if culturally appropriate. In the focus groups, most professional stakeholder participants said they would not think to ask for an interpreter without prompting, yet they were perceived favorably, although in short supply. They further reported that most specialists they knew did not use the health interpreter service available and interpreters were not used by general practitioners given different funding streams. One participant spoke of a female specialist who always books an interpreter for patients from non-English speaking backgrounds, regardless of accompaniment of a family member:

*There wasn’t always enough [interpreters]. Um, it depends on the language, too; some are hard to get. Maybe Turkish or something like that might be hard to get. But we had their [phone] numbers and we certainly used to ask if they wanted an interpreter, because we didn’t like their family accompanying them in to the doctor, because a lot of those women wouldn’t want to say various things in front of their children, for example, and – and not their husbands, so that would be hopeless. So we used to ask if they wanted an interpreter and we would make the interpreter’s appointment at the same time, if we could get them. (CM)*

Furthermore, it was expressed that most informational resources are in English which makes it very hard for carers from CALD backgrounds to navigate the health system without support. Additional challenges faced by older women who migrated many years ago reflect differences in meanings and norms across cultures and generations. One participant described these groups as particularly at-risk:

*“People who are stuck, like, they migrated 30 years ago and the cultures over there have changed, but they haven’t. They’re, sort of, stuck in that patriarchal, sort of, mindset.” (PS)*

In addition to assumptions of English language proficiency, health professionals assume the nomenclature around ‘chronic conditions’ is relevant and accessible to all. One participant highlighted that this language and its meanings are not necessarily relevant to their CALD communities:

*“I think the whole idea of chronic health conditions is so Anglo, I’m really sorry to say…Instead, women from different cultural communities adapt to having what we term as ‘chronic conditions’ and don’t identify as such…they just seem to get on with it, and they do really just put themselves second. Um, I know that’s an old adage, but I think if you add the cultural layering around it, there’s a sense of, um, stoicism in some cultures.” (PS)*

Some women do not identify as having ‘chronic conditions.’ Women may adapt to changed capacity for physical function or symptom experiences rather than constantly attending to them and labeling them as illnesses. This participant goes on to attribute such adaptation to a cultural characteristic of stoicism, such that it is not deemed appropriate, or a norm, in a given society to express need, pain, or other vulnerability.

### Social and economic constraints and caregiving roles exclude women from participating in self-care and society

The disproportionate burden of caregiving on women impacts negatively on self-care. Participants discussed that policies of early discharge from hospital lead to more demands on informal caregivers who must continue care coordination outside hospital without formal assistance. They noted that caregiver women were less likely to look after themselves, had less time to themselves, and experienced more stress and pressure to meet competing demands. Caregivers often have chronic conditions, yet these were less prioritized in their, and in some cases, other people’s views. Participants noted that women may accompany a male partner to a medical appointment, potentially offering advocacy, but the reverse was less common. Generally, women were seen to be more proactive than men in seeking support for others, but often delayed until crisis point for themselves because they did not want to be a burden.

Participants reported that a low level of caregiver awareness, wherein women did not identify as a caregiver, was common. Many women do not acquaint support of partners and family members as an additional role, but rather, inherent within maternal, spousal, or friendship roles. Cultural beliefs, age, and generational characteristics also contributed to beliefs around caregiving. It was noted that Aboriginal women tended to take on a greater burden of caring in their extended community and often contribute significantly to caregiving for grandchildren. Women’s lack of caregiver awareness has implications for their accessing outreach services in mainstream settings. One participant shared her perspective of the impact of the lack of support for women caregivers in the community:

*“Women in the community would see that they’re being left to their devices. I think there’s very little demonstrated outreach. Who touches these women? Where do they get a sense of being part of the society in that, you know, who looks after them? So, the message they’re constantly getting over the years, and as long as you've been a carer, there’s this disempowerment… no one is reaching out, really. Or there’s barriers, or hoops they have to go through, like, funding.”(PS)*

The caregiver journey was perceived as an isolated, complicated, and unsupported one. Another community member participant felt that women need to be encouraged to practice self-care, although many are not accustomed to doing so given their roles as caregivers:

*“…that’s a problem for a lot of women that they’re always looking after someone else and they – they don’t look after themselves…I think women enjoy, very much, that aspect of that personal touch, the feeling of being looked after…these women are only over 50 or 55 so, they’re not used to doing what they want to do when they want to do it.” (CM)*

High costs of specialist and other appointments as well as associated transport, parking, and meals contributed to financial hardship for women. Caregiving and having a chronic condition impacted on workforce participation and subsequent income. It was explained by participants that many women did not drive and were dependent on family members for transportation. Women can be socially excluded with few transport options which can lead to feelings of isolation.

*“There’s no way out….yeah, you’re gonna be locked in this house. If you don’t drive, you know, you can’t go anywhere…You got the buses, maybe the bus every three hours they go somewhere, so what are you going to do?” (CM)*

*“There’s a lot of ladies that want to come and join this group but they live further away. They don’t drive.” (CM)*

*“working in the communities with the groups, yeah, there is quite a few old people on their own… who I see in the centres, but there’s a lot ladies that are in the community that just don’t go… And they’re the ones that get lost, and end up leaving the house, or bouncing in and out of hospital.” (PS)*

In cases where there is intimate partner (or other domestic) violence, which was described as under-reported in the study region and represented a significant deficit in relation to needed supportive services, also had carried financial implications for women thinking of leaving a violent relationship.

*“More likely to be poverty stricken if you go it on your own” (PS)*

An important consideration is that not everyone in need meets the eligibility requirement for formalized support and services. Participants shared that women caring for children and fleeing from violent relationships face disadvantage as they or their children do not always qualify for protection. Not all temporary housing options are safe places. Expenses associated with transportation to and from refuges, and other services and lack of readily available transport options were many. Charities no longer have the resources to assist with subsiding transport fares and prescriptions. Women who receive waivers from a charity are often cut off after a period of prolonged affiliation. When appointments were available, transportation was generally described as problematic.

*“If we do a referral to women’s health, what is available for them? Can they get there? How soon?…After-hours hotline is given as an option; have you got bus fare to get to a refuge?” (PS)*

*“maybe there’s a car in the family, but [the women] probably don’t have it.” (CM)*

### Integrated networks that facilitate voice and enable communication and engagement empower women

Ensuring understanding and facilitating communication are essential to women’s empowerment, as explained by one participant:

*“Women really need to be empowered, and we all know that people feel comfortable in their first language, especially when they’re older, because people regress to their culture. And with women, word of mouth becomes so central with the way they live their lives, with women, other women on the phone, or at the shopping centre… It’s this verbal, and I think if you cut that off, you’re not giving women the tool that they use.” (PS)*

Likewise, within health service facilities and outreach initiatives, health professionals who appreciate the importance of getting a sense of who the person is and what they need and want is vital to providing person-centered care. Increasing capacity for cultural sensitivity in providers was referenced.

*“for a nurse to come home. Someone that has cross-cultural and spoke another language, but didn’t necessarily speak that language, willing to use interpreter and - just see what her needs are what her world is, get a picture of her world, in her language, in her understanding of how it would be, and her socio-economic level, a picture of her world…” (PS)*

Professional stakeholder participants discussed that women they worked with, particularly from new arrival and refugee communities preferred face-to-face outreach and female health workers/professionals. Some cultural groups were described as having expectations of community outreach as opposed to in-reach or attending a specific physical premises or doctor’s surgery to receive care. They also preferred the worker/health professional to be from their own culture or at least culturally sensitive. Although home visits by nurses were appreciated, socioeconomic disparities would act as barriers if ‘Anglo’ nurses were doing home visits. Cultural stoicism was also cited as a barrier to home care outreach:

*“Sometimes it’s stoicism around that, from a cultural point of view; where I don’t want to show that I’m not looking after my house and making my meals.” (PS)*

Building up of trust through sustained functional relationships over a long period of time was described as necessary to engaging women in communities. Community-based events or venues were reported to be effective in engaging women:

*“I mean, those opportunistic outreach opportunity - like the shopping centres, community events or just community thoroughfares, those places where women frequent, I think having a presence, an appropriate presence by the health service…There’s some work around mobile services in disadvantaged communities, which have been successful for women.” (PS)*

An example of community health programs integrated with other initiatives to engage refugee women was discussed as having positive and negative features. The refugee health program linked women to a general practitioner, English classes, opportunities to increase health literacy, and information on reproductive health which was important to these women. Unfortunately, not all resources were in their language.

A local women’s-only health facility was perceived as a safe space. This facility did not allow men into its premises and did not allow husbands, boyfriends, or children to accompany women. Participants explained that when a previous women’s health clinic opened in the area, some CALD women reportedly couldn’t believe it was a service dedicated to women, as they had never encountered the prioritization of women in their home countries.

*“So it was a – a man free place. And that made a huge difference, you know, because a lot of women who came there were women who’d been beaten up by men…We got a lot of those, although we weren’t funded for domestic violence – that didn’t stop women coming in, you know, bruised and beaten and asking for help…Especially in that area, but probably all over the [this region].” (CM)*

Initiatives to build social capital and networks in communities were discussed as a way of ensuring women did not slip between the cracks of service provision. For example, older women living alone in the community are often overlooked service-wise.

*“If women have a good group around them, like neighbours and friends that will help. It doesn’t have to be a big group, just some key important people that, ah - all women should look out for them that way, that’s this mutual thing.” (PS)*

*“Just by the nature of the way women, and the strengths that women have, I think that I’ve seen in the CALD communities, women use the verbal; they - they want word of mouth, they have trust amongst their informal networks.” (PS)*

#### Health professionals play a key role in gender-based approaches and negotiating the system

Professional stakeholder participants discussed their positions within the health system as an asset in that they can advocate to ensure women’s health needs are being met.

*“Health workers have to speak up for rights of women even in their own workplaces.” (PS)*

They described how they used their position, organizational and systems knowledge, and networks to acquire services for clients. It was the professional relationships that were very important to getting access.

*“If you go thought the front desk, you’ll never get services for anyone. If you know someone behind the scenes, you can use it to your advantage.” (PS)*

Despite these advantages, professional stakeholders likewise shared perspectives of systems-level barriers to gender-based health service provision stemming from lack of integration of services and communication. Services addressing only discrete issues and working in specialties and sub-specialties that do not communicate with each other added a high degree of complexity and inaccessibility to health care.

*“And there has to be a funding model where community support services are able to be funded as part of the health service, not separate. And, I think, outsourcing to NGOs (non-government organizations) is a good thing, in that you’re empowering and building up the community sector, where do women mostly get their supports? - From the community sector. But there has to be a real link between both. So primary, secondary, tertiary care can only work well with a really supportive community sector.” (PS)*

For older women, in particular, who often have multiple chronic conditions, but whom are impacted by social, cultural, sometimes linguistic, and economic contexts that impede care, the silo-structure is a major obstacle. It was noted that although policy reflects the ideal of holistic care, it was expressed that workforce time constraints and structural inconsistencies mean holistic care remains an aberration.

A recurrent topic was the phenomenon of building up consumers’ expectations for support, but failing to deliver. This failure impacts on health providers who are on the front line and often face the anger of disappointed consumers. This leads cynicism and loss of trust within the workforce, as well as in consumers, as illustrated in the following excerpts:

*“I hear all the time of carers try getting domestic assistance to come and get it. Because you do, you ring up, and no one would call, or they’ve got a waitlist - - - some don’t even have a wait list, or then the social worker told me, ‘Oh, you've got to ring every month.’ So, it’s like a big hassle, you know. You may as well clean the house yourself for an hour, rather than sit on the phone for three hours ringing all the service providers.” (PS)*

*“People don’t understand about respite, what that even means. And even - I mean, in a way we set up false expectations, because we go in and tell them, ‘Oh, there's all these services available.’… And then they ring, and there aren’t all these services available.” (PS)*

Although these service deficiencies impact directly on quality of life for older people and carers, in particular, the erosion of the health workforce morale is collateral damage.

## Discussion

A prominent and concerning theme that emerged in this study involved the disempowerment of women in interactions and communication with health professionals. These findings corroborate previous research in women’s health issues wherein women have expressed feeling dismissed and invalidated in encounters with health professionals [12], stereotyped [23], or not having their health care needs met [23]. Consequences of such disempowerment can lead to exacerbations of health problems due to avoidance and delayed care seeking.

The lack of understanding due to language differences and use of medical terminology stifled women’s engagement in their health management. Health professionals may erroneously assume satisfactory health literacy levels and language proficiency, particularly in women who have had longstanding chronic conditions or who were caregivers [24]. Our data also highlighted the additional challenges faced by ageing women from CALD backgrounds. For instance, CALD women with chronic conditions may feel separated or isolated due to language or other socio-economic barriers [25] or may find it difficult to identify with mainstream portrayals of women depicted in health campaigns, thus influencing self-management [18]. Inconsistent use of interpreters highlights a need for health professionals to be trained in working with interpreters [26–28].

The experience of caregiving was likewise described as disempowering for women with chronic conditions given the lack of outreach services. The data corroborated previous research that has found caregiving responsibilities for a partner or elderly parent inhibited participation in secondary prevention programs, for example [29]. The low level of caregiver awareness, or self-identification, meant that many did not attempt to obtain support. The National Carer Strategy (2010–2014) [30] was the Australian government’s initiative to ensure recognition and respect, information and access, economic security, services, education and training, and health and wellbeing for carers. Based on perspectives of some of the professional stakeholder participants, practical elements of this initiative may not yet have been realized by many women in this community.

Intimate partner violence was described as a significantly under-reported phenomenon in the area. As has been called for in low and middle-income countries, initiatives to integrate responses to intimate partner violence are needed in the health sector in the Illawarra region [31].

The impact of systems failures and insecure funding of programs and positions on health services personnel cannot be discounted [32]. Morale corrodes upon repeatedly witnessing the impact of inadequate and inconsistent funding on individuals and communities. Our data showed the important advocacy and networking role played by many health professionals and front-line staff.

Results of the focus group study endorse 1) development of programs for CALD women within a framework of cultural beliefs and consideration of gender issues, 2) using interpreters, bilingual health workers/community educators, 3) facilitating a welcoming, comfortable, and safe environment, 4) developing and sustaining partnerships and networks, and 5) offering respect, flexibility, and responsiveness, mindful of varied contexts and complexities not amenable to population-level approaches.

Although gender is of critical importance in promoting access to health care, it is not commonly addressed in mainstream services. As the health care system shifts the focus to more primary and community care based services addressing the specific need of women is of critical importance. In this report of barriers and facilitators to providing a gender-based approach to women's chronic conditions, we have described socio-cultural considerations and provided descriptions of women's interface with the health care system. Our findings have underscored the need to tailor programs to meet the unique needs of women.

### Limitations

Additional perspectives can likely inform this topic, particularly from different groups of women within the community. Future recruitment efforts should extend to additional community groups and non-government organisations and data collection should take place within these settings. Aboriginal women were not targeted in this data collection phase. Additional group discussions should be scheduled to speak with women and health workers/professionals within the Aboriginal communities of the study region as their perspectives and experiences would add significant depth to our understanding. Interpreters were not available on data collection dates, so the views of additional CALD women who did not speak English are absent from this report. Fewer participants attended the focus groups than agreed to participate which resulted in a small sample size. We were advised that non-participation in this study reflected incongruous work schedules for stakeholder participants and lack of transport for some community member participants. Efforts were made to hold multiple groups over a two month period in varied locations to facilitate access. Although this is a known challenge of focus groups, additional strategies will be required to facilitate future recruitment. A key strength of this study was the ability to hear from women and professional stakeholders from the targeted community. Their perspectives added depth and real world examples grounded in the contemporary Australian context; an integral part of intervention development.

## Conclusions

This project underscores the importance of including both perspectives of sex and gender in health care services planning. Tailoring and targeting of health care services to particular socio-demographic and cultural groups is critical in promoting access to health care services. Unique epidemiological trends, particularly the ageing of women and new migrant groups requires particular attention.

A range of social, cultural and economic factors are rapidly changing the landscape of health care. Social ecological frameworks have been widely adapted with the recognition that no single factor can explain or predict a particular phenomenon. This requires the systematic examination of the health care system to address the barriers and enablers to improving health care for women. A social-ecological framework is likely useful to frame the discussion, debate and formulation of interventions to improve the health care of women. This use of this approach identifies a framework for improving health care as the outcome of an interaction among a range of factors. Looking at the intersecting relationships between the individual, interpersonal relationships, health care organizations, community groups and public policy are critical in promoting gender based initiatives to improve the health outcomes of women.

## Abbreviations

CALD: Culturally and linguistically diverse
CM: Community member participants
PS: Professional stakeholder participants
GBA: Gender-based approach
NGO: Non-government organization
